# The financial need of feeding infants for the first six months of life in West Java Province of Indonesia and the implications of socioeconomic and mental health factors

**DOI:** 10.1186/s13006-023-00561-5

**Published:** 2023-05-15

**Authors:** Riki Relaksana, Adhadian Akbar, Estro Dariatno Sihaloho, Dani Ferdian, Adiatma YM Siregar

**Affiliations:** 1 Center for Economics and Development Studies, Department of Economics, Faculty of Economics and Business, Universitas Padjadjaran, West Java, Indonesia; 2 The Task Force of the Acceleration of Stunting Reduction, The National Population and Family Planning Board (BKKBN), West Java, Indonesia; 3 West Java Development Institute (INJABAR), Universitas Padjadjaran, West Java, Indonesia; 4 Department of Public Health, Faculty of Medicine, Universitas Padjadjaran, West Java, Indonesia; 5 Center for Health Technology Assessment (CHTA), Universitas Padjadjaran, West Java, Indonesia

**Keywords:** Breastfeeding, Costing, Depression symptoms

## Abstract

**Background:**

In Indonesia, nearly half of all children aged less than six months were not exclusively breastfed in 2017. This study aimed to compare the cost of providing direct or indirect exclusive breastfeeding 0–6 months, partial exclusive breastfeeding and commercial milk formula only. This study also assessed the maternal socioeconomic and mental health factors to providing exclusive breastfeeding.

**Methods:**

Data were collected in 2018 via a cross-sectional survey of 456 mothers in Bandung City and Purwakarta District, West Java Province, Indonesia, who had children aged less than six months. We used micro-costing to calculate the cost of productivity, equipment, supplies, and training of mothers when providing direct exclusive breastfeeding, indirect exclusive breastfeeding, partial exclusive breastfeeding (a mix of breastfeed and commercial milk formula), and infant formula/commercial milk formula only. Logistic regression was used to determine the impact of several independent variables, including mother’s level of depression, on exclusive breastfeeding.

**Results:**

To provide direct exclusive breastfeeding, the cost per mother in the first six months is US$81.08, which is less expensive than indirect exclusive breastfeeding (US$171.15), partial exclusive breastfeeding (US$487.8) and commercial milk formula (US$494.9). We also found that education and age are associated with the decision to provide direct exclusive breastfeeding. Mothers who work will most likely provide indirect exclusive breastfeeding, commercial milk formula, or partial breastfeeding as opposed to direct exclusive breastfeeding. Finally, although severe depression symptoms have a positive relationship with the decision to provide commercial milk formula over direct exclusive breastfeeding, the evidence here is not strong.

**Conclusions:**

The total cost of providing only commercial milk formula is 6-times higher than the cost of direct exclusive breastfeeding. The presence of severe depression symptoms is positively related to mothers opting for other feeding methods aside of direct exclusive breastfeeding and indirect exclusive breastfeeding. This study shows that direct exclusive breastfeeding is economically preferable to other methods, supports policies to reduce the time cost of exclusive breastfeeding (e.g., paid maternity leave and maternal cash transfers), and addresses the importance of mother’s mental health to ensure successful breastfeeding.

## Background

Indonesia has the potential to increase its productivity by 2045 due to the low dependency ratio and high productive labor force (age 15–64 years), better known as a demographic bonus [1, 2]. In order to achieve this, issues such as children’s health should be addressed, as children are crucial in realizing the demographic bonus. However, as of 2018, Indonesia still had prevalent issues concerning children’s health, such as underweight, wasting, and particularly stunting, which affected 30.8% of hildren aged 0–59 months [3]. Stunting is associated with a reduction in schooling years, risk of grade failing, more unsatisfactory cognitive performance, and lower school achievement [4, 5]. Thomas and Strauss [6] found that an increase in height of 1% is associated wth a 2.4% increase in wags in Brazil. According to UNICEF conceptual framework on maternal and child nutrition, breastfeeding has a crucial role as an underlying determinant of the short- and long-term outcomes for childhood, adolescence, adulthood, and societies (e.g., improved survival, health, physical growth, productivity, cognitive development, wages in adults) [7]. Breastfeeding newborn babies is crucial for the physical and mental health of both the mother and baby [8], and it can reduce the mother’s depressive symptoms [9]. Subsequently, support from the government would have considerable benefits [10].

Unfortunately, only half of children under six months of age in Indonesia were exclusively breastfed in 2017 [11]. Although this has surpassed the national strategy target of 50% o children under 6 months being breastfed exclusively, this still does not reach the global breastfeeding target of 70% b 2030 [12, 13]. Factors such as mental health and socioeconomic factors may hinder the success of exclusive breastfeeding [14, 15].

Several studies have found that approximately 10% of pregnant women and 13% of women who recently became mothers experience a mental disorder, primarily depression and anxiety [16, 17], which influences the success of exclusive breastfeeding [18, 19]. Social norms, body image, convenience, perception of commercial milk formula (CMF), difficulty breastfeeding, and lack of confidence hinder the provision of breastmilk and may lead to the use of CMF [20]. Moreover, massive marketing of CMF could affect the decision of the mothers to breastfeed [21].

The impact of the decision to not breastfeed also has economic consequences. As the country with the lowest exclusive breastfeeding rate among the Association of Southeast Asian Nations (ASEAN), Thailand has experienced a loss as high as 0.5% of their gross national income (GNI) [22]. The effects of not breastfeeding, such as diarrhea, pneumonia, and cognitive loss, will also lead to considerable economic cost [23–27]. In Indonesia, this cost can reach US$1.5–9.4 billion annually, and the annual number of maternal and infant deaths can reach more than 7000 [10, 22, 28].

West Java is the most populous province in Indonesia, with approximately 48.2 million residents in 2020 [29]. Among children aged 0–59, 25.7% i West Java experience stunting [30], but 63.5% of chldren < 6 months old were exclusively breastfed in 2019 [12]. Though both rates are better than the rates at the national level, the large population in West Java basically results in a higher magnitude of problems in terms of both health and economic impact. For example, the economic impact of not breastfeeding due to respiratory diseases and diarrhea in West Java (US$26.7 million) is more than 5-times higher than that of North Sumatera, the fourth most populous province in Indonesia (~ 15 million people; US$5.9 million) [28].

To the best of our knowledge, the financial need of mothers to provide breastmilk and CMF has rarely been studied, especially in the context of the ASEAN and Indonesia. In this study, we estimated the financial need of mothers who provide breastmilk (either directly or indirectly through feeding expressed breastmilk), commercial milk formula, or a mix of both and explore the factors that may influence this decision, namely socioeconomic factors and mental health. The results will be useful for policy makers to better understand the behavior of mothers in making decisions about breastfeeding and aid in developing proper policies to further promote breastfeeding.

## Methods

### Study design

We divided our samples into four groups: those who directly exclusively breastfeed (DBF), those who indirectly exclusively breastfeed (IBF), partial exclusive breastfeeding (PEB; a mix of breastfeeding and CMF), and those who provide only commercial milk formula (CMF). We used a micro-costing approach to calculate equipment cost, supplies cost, and productivity loss in the respective groups [31]. We also calculated the cost of training and consultation for mothers who DBF, IBF, or PEB. We calculated our cost within the time frame of six months.

### Study setting

We collected data from Bandung City (the capital of West Java province) and Purwakarta district in 2018. During the study period, Bandung City had the highest rate of six months exclusive breastfeeding in West Java (67.3%), whereas Purwakarta district had the lowest (55.1%) [32]. Based on sample size estimation using the 95% cnfidence level, we needed at least 210 mothers with children under six months old in each region. In the end, we surveyed 456 mothers with children < 6 months old.

### Data collection

We provided the respondents with pictures of 25 types of equipment and supplies for breastfeeding and CMF and asked them to choose the relevant pictures of what they used. We estimated the market prices from several websites to obtain the average price. The equipment cost was annualized to obtain yearly cost. Table 1 provides a detailed list of equipment and supplies, as well as their estimated price and the percentage of respondents who own and utilize the goods. We obtained the list of equipment and supplies based on a reference from a previous study [33]. We summed up the total equipment, supplies, and training cost for all respondents from each group and divided it by the number of respondents in the group to obtain the direct cost per mother.

**Table 1:** Supplies and equipment used by respondents

Breastmilk or both CMF and breastmilk						CMF or both CMF and breastmilk			
	Price ($)	% of mothers who own the equipment					Price ($)	% of mothers who own the equipment	
		DBF^1^	IBF^2^	PEB^3^				CMF^4^	PEB
Nursing bras	3.53	35	56	52		Baby Bottle	1.9	89	76
Nightshirts	9.94	79	71	54		Teat	0.6	85	77
Breast pads	0.08	1	29	5		Sterilizer	16.1	4	5
Antiseptic nipple spray	1	1	12	2		Formula milk (800 g)	16.1	70	67
Breast cream	16.45	1	21	5		Bags	11.3	41	29
Refrigerator	122.23	0	50	20		Bottle warmer	7.8	11	8
Nipple shields	7.92	0	15	2		Powder dispenser	3.7	56	39
Breast pump	10.32	0	50	6		Bottle brush	1.4	56	49
Breastmilk bags	11.29	0	29	7		Baby bowl	1.3	81	75
Breastmilk storage bottles	10.2	0	47	2		Stove	20.9	93	78
Sterilizer	16.08	0	12	1		Thermos bottle	6	74	70
Support pillow	6.19	1	21	1		Pan	3.4	85	64
Ice gel	0.87	0	26	1		Cup feeder	1	4	7
Freezer	88.48	0	9	0		Blender	31.4	37	52
Breast shells	11.66	0	9	1		Food processor	243.7	4	7
Stove	20.85	0	65	23		Food filter	1.1	26	49
Pan	3.45	0	62	23		Infant cup bottle	11.9	7	11
Thermos bottle	6.02	0	65	24		Ice cube mode	2	0	29
Bottle warmer	7.85	0	24	1		Juice extractor	29.6	4	3
Baby bottle	1.93	0	76	26		Baby chair	16.4	7	17
Teat	0.55	0	68	26		Bottle cleanser	1	48	46
Bottle brush	1.39	0	59	17		Plate	7.3	33	56
Cup feeder	1.05	0	15	6		Spoon	0.5	81	74
Bottle cleanser	1.04	0	68	13		Slow cooker	18.8	11	1
Baby carrier	2.6	73	88	54		Streamer	19.5	4	1

Direct exclusive breastfeeding, ^2^Indirect exclusive breastfeeding, ^3^Partial exclusive breastfeeding, ^4^Commercial milk formula

Productivity loss was calculated based on the income per household adjusted for six months divided by the estimated work minutes per six months to find income per minute. The number was multiplied by the time needed to prepare and provide breastmilk and/or CMF. More specifically, for the DBF group, this involves the time needed to breastfeed, while for the IBF group this involves the time needed to express breastmilk (either by hand or by using commercial breast pump), prepare and sterilize equipment, and to feed the breastmilk. For the CMF group, this involves the time needed to prepare and sterilize equipment, prepare the CMF, and to feed the milk, while for the PEB group this involves the combination of the other groups’ use of time. In addition, for mothers in DBF, IBF, and PEB groups, we also calculated the time to receive training and/or obtain consultation regarding breastfeeding and the process to express breastmilk, as well as any relevant preparation. We did not calculate such training/consultation for the CMF group as none of our respondents in this group received such training/consultation. For each group, we totaled the productivity loss of all households and divided it by the number of households per group to obtain the productivity loss per mother for each group.

### Data analysis

We also carried out multinomial logistic regression to determine factors that may influence the decision on how to provide breastmilk or CMF. We used the same categories as with the costing analysis for the dependent variable (i.e., DBF, IBF, PEB, and CMF). The socioeconomic variables (e.g., age, marital status), time needed to provide infant formula and/or CMF, cost and time spent to prepare infant formula and/or CMF, and depression symptoms were used as independent variables. Table 2 provides the complete list of variables used for the logistic regression.

**Table 2:** Variables used for multinomial logistic regression

Variable	Description	Category	Value
$kind$	Type of feeding	Direct exclusive breastfeeding (DBF)	1
		Indirect exclusive breastfeeding (IBF)	2
		Partial exclusive breastfeeding (PEB)	3
		Commercial milk formula (CMF)	4
$work$	Employment status	Working	1
		Not working	0
$mar$	Marital status	Married	1
		Divorced	0
$educ$	Education	Senior high school or higher	1
		Middle school or lower	0
$age$	Age in years	Average	29
		Min-Max	16–54
$child$	Number of children	Average	2
		Min-Max	1–5
$time$	Time required to breastfeed/provide CMF in minutes	Average	188
		Min-Max	14-1200
$dep$	CESD-R-10*	Average	-2.44
		Min-Max	-5.95-0.89

*CESD-R-10, Center for Epidemiologic Studies Depression Scale – Revised

This table shows the variables used in multinomial regression analysis

## Results

### Respondent characteristics

Among the 456 respondents, 310 provide DBF, 32 IBF, 87 PEB, and 27 CMF; 32% of the respondents did not provide DBF (Table 3). More than half of the respondents from each group were < 30 years old, but most respondents had completed education up to senior high school. Almost all respondents were married, and most of them were not working and had more than one child.

**Table 3:** Respondents’ characteristics

	DBF			IBF^2^			PEB^3^			CMF^4^	
	n	%		n	%		n	%		n	%
*Age*											
< 30 years	188	61%		19	59%		45	52%		16	59%
> 30 years	122	39%		13	41%		42	48%		11	41%
*Education*											
Did not go to school	2	1%		0	0%		1	1%		0	0%
Elementary	52	17%		4	13%		13	15%		8	30%
Junior high school	131	42%		4	13%		25	29%		5	19%
Senior high school	109	35%		12	38%		40	46%		12	44%
Diploma	9	3%		2	6%		2	2%		0	0%
Undergraduate	7	2%		10	31%		6	7%		2	7%
*Marital status*											
Married	304	98%		32	100%		83	95%		25	93%
Divorce	6	2%		0	0%		4	5%		2	7%
*Work status*											
Employed	14	5%		11	34%		10	36%		24	28%
Unemployed	296	95%		21	66%		18	64%		62	72%
*Number of children*											
1	109	35%		12	38%		36	41%		14	52%
> 1	201	65%		20	63%		51	59%		13	48%

Direct exclusive breastfeeding, ^2^Indirect exclusive breastfeeding, ^3^Partial exclusive breastfeeding, ^4^Commercial milk formula

### The financial need of providing milk

The highest average cost of equipment used per mother for the first six months is found within the PEB group (US$ 104.12 per six months), followed by IBF, CMF, and DBF. The lowest average supplies cost per mother is found in the DBF group (US$0.30 per six months), equivalent to only 0.4% of its average total cost. Average CMF cost dominated the supplies cost in the PEB and CMF groups. Table 4 presents the cost breakdown for each group.

**Table 4:** Cost components per mother over 6 months

Component	DBF	%	IBF^2^	%	PEB^3^	%	CMF^4^	%
**Productivity loss**	US$13.02	18.0%	US$43.58	31.6%	US$24.03	5.1%	US$35.97	7.8%
**Equipment**	US$54.55	75.2%	US$67.35	48.9%	US$104.12	22%	US$49.88	10.9%
**supplies**	US$0.30	0.4%	US$13.21	9.6%	US$339.17	71.6%	US$372.46	81.3%
**Training and consulting**	US$0.18	6.5%	US$13.7	9.9%	US$6.06	1.3%	n/a	0%
**Total**	US$81.08	100%	US$171.15	100%	US$473.38	100%	US$458.31	100%

Direct exclusive breastfeeding, ^2^ Indirect exclusive breastfeeding, ^3^ Partial exclusive

breastfeeding, ^4^Commercial milk formula

### Productivity loss

As seen in Table 5, the time spent per day to provide infant formula and/or CMF to infants is the longest in the CMF group (420 min) and the shortest in the DBF group (167 min). Converting these times into the productivity loss over six months showed that IBF cost has the highest productivity loss (US$ 43.58/mother/6 months) due to the highest productivity loss per minute (Table 5).

**Table 5:** Average time spent related to providing infant formula and/or CMF per day (minutes) and average productivity loss (US$) per mother over 6 months

	Average time spent per mother (minutes) per day					Average productivity loss per mother (US$)		
	Sterilization/ warming	Pumping/ preparing	Feeding	Total time spent		Per minute	Per day	Per 6 months
DBF	-	-	167	167		0.0004	0.07	13.02
IBF^2^	41	38	323	402		0.0006	0.24	43.58
PEB^3^	21	52	240	313		0.0004	0.13	24.03
CMF^4^	41	39	340	420		0.0005	0.19	35.97

Direct exclusive breastfeeding, ^2^ Indirect exclusive breastfeeding, ^3^ Partial exclusive breastfeeding, ^4^Commercial milk formula

### Multinomial logistic regression

Table 6 presents the multinomial logistic regression results. Mothers who work are most likely to provide breastmilk or milk through IBF, PEB, or CMF. In terms of costs, those who provide IBF, PEB, or CMF are most likely to incur larger costs. Lastly, it seems that mothers who experienced severe depression symptoms may potentially provide DBF, except for mothers in the IBF group, although this relationship is weak.

**Table 6:** Multinomial logistic regression analysis showing Relative Risk Ratio (RRR)

	IBF^1^	PEB^2^	CMF^3^
*CESD*			
RRR^4^	0.911	1.049	1.032
P value	0.530	0.909	0.943
CI^5^	0.679–1.219	0.457–2.410	0.427–2.490
*Work*			
RRR^4^	1.0001	1.0004	1.0004
P value	0.001	0.000	0.001
CI^5^	1.00007–1.0002	1.0001–1.0006	1.0001– 1.0006
*Total Cost*			
RRR^4^	1.000002	1.000005	1.000005
P value	0.000	0.000	0.000
CI^5^	1.000001–1.000003	1.000003–1.000007	1.000003–1.000007
*Education*			
RRR^4^	2.567	0.162	0.072
P value	0.099	0.154	0.086
CI^5^	0.837–7.868	0.013–1.984	0.004– 1.452
*Children*			
RRR^4^	1.575	1.275	0.328
P value	0.445	0.866	0.491
CI^5^	0.491– 5.054	0.075–21.572	0.014–7.831
*Age*			
RRR^4^	1.010	0.875	0.904
P value	0.806	0.185	0.342
CI^5^	0.928–1.1003	0.719–1.065	0.735–1.112
*Marriage*			
RRR^4^	1.001	0.999	0.999
P value	0.991	0.644	0.523
CI^5^	0.782–1.283	0.999–1.0006	0.999–1.0005

^1^Indirect exclusive breastfeeding, ^2^Partial exclusive breastfeeding, ^3^Commercial milk formula, ^4^Relative Risk Ratio, ^5^95% Confidence Interval

## Discussion

Our results show that mothers in the direct exclusive breastfeeding group bear an approximately 6- times lower cost of providing breastmilk in the first six months than mothers in the partial exclusive breastfeeding and commercial milk formula groups. The cost drivers of direct exclusive breastfeeding and indirect exclusive breastfeeding mothers are productivity loss and equipment, whereas the cost driver of mothers in the PEB and CMF groups is mainly commercial milk formula. Although productivity loss is one of the cost drivers for the DBF and IBF groups, the nominal amount is roughly similar to the productivity loss found in the PEB and CMF groups. As the productivity loss of DBF group consists only of direct breastfeeding, it has the lowest productivity loss (and the average total time spent to breastfeed), as the other groups require additional time to either express breastmilk, prepare and sterilize equipment, and/or prepare CMF, in addition to the time needed to feed the infant formula and/or commercial milk formula. Added by the high cost of CMF, the cost of providing infant formula and/or CMF per mother in the first six months of the PEB and CMF groups is much higher than the DBF and IBF groups. Similarly, a study in England found that, although IBF require purchasing equipment before the babies were born (e.g., this includes breast pumps, breast-milk freezer bags, muslin cloths, nipple shields, breast shells, breast and nipple creams/sprays, breast-milk storage bottles, sterilizers and support pillows), the cost of providing CMF is still 62% more expensive than IBF [33]. Such huge cost difference should be shared with the public more frequently to show the potential savings if a mother decides to DBF or IBF. Unfortunately, such information is rarely available, especially in developing countries, and should be studied more in different settings and socio-economic groups.

Regardless of the type of milk, the opportunity cost of providing infant formula and/or CMF has been recognized as one of the main challenges in breastfeeding, as mothers need to spend time providing, preparing, or expressing the breastmilk and leave their work for a certain amount of time, which is viewed as causing productivity loss [34]. No policies in Indonesia are currently in place that directly address such views and challenges. Considering this finding, a careful assessment of the need of mothers, firms/institutions, and other stakeholders in achieving and supporting optimal breastfeeding conditions should be conducted to design appropriate policies.

We also found that mothers who experience severe depression symptoms are potentially more likely to provide PEB or CMF than DBF. Although this relationship is weak, newer and older studies support the association between breastfeeding difficulties and depression [35–37]. This aspect is rarely discussed in developing countries and requires specific procedures and policies. However, this requires further studies as we currently do not have sufficient local evidence to support this argument.

Lastly, in all groups, mothers who work have a slightly higher chance to opt for either IBF, PEB, or CMF, instead of DBF. This can be partly explained by the not optimal or the absence of maternity support at the workplace [38]. Indeed, the existence of workplace interventions to support breastfeeding is crucial to increase the duration of breastfeeding and avoid early introduction of CMF [39]. This is important, as, in the case of urgent need of income, mothers will most likely hurry to get back to work. Although regulations supporting maternal leave are already in place in Indonesia, they only cover the primary salary, and additional income-benefits associated with mothers’ work are not given during maternity leave [40]. This would become an issue, especially in the case of a larger benefit compared to the primary salary. As such, mothers face hard choices between taking the time to DBF or getting back to their office as early as possible without utilizing the full length of their maternity leave [41–43]. Moreover, we also should note that although the cost of DBF in the first 6 months is 6 times lower than the CMF group, it is not free. There are still existing opportunity and equipment costs, and such costs may discourage mothers from breastfeeding if informed incorrectly without proper explanation of the costs of other feeding options (i.e., IBF, PEB, CMF). Given these challenges, optimizing maternity protection policies is crucial.

Studies have shown that extending maternal leave in the formal and informal sectors is an economically attractive option, but other supporting policies, such as maintaining income at the appropriate level, flexible working hours, the existence of a lactation room, will also encourage mothers to DBF at the workplace [44–47]. In addition, reimbursement policies may also provide benefits to the DBF rate. One such example is requiring employers or insurance policies to cover or reimburse the costs of prenatal and postnatal lactation support, counseling, and equipment rental during breastfeeding period [48]. Another form of reimbursement is to share the financial need of paid maternity leave between employers and government, in which the government reimburses a certain % of the financial need already spent by the employers to pay for e.g., salary of mothers during paid maternity leave, thus reducing the burden of employers [44, 49].

Additionally, mothers’ education seemed to be associated with mothers’ decision to provide DBF and/or CMF, and mothers with higher education are most likely to provide DBF instead of CMF. Thus, encouraging women to obtain education may increase the rate of mothers to DBF [50], although it may also lead to a negative effect as there is higher probability of mothers to return to workplaces where support for breastfeeding is limited [51]. This requires similar attention as discussed in the previous paragraph regarding breastfeeding at the workplace.

This study has some limitations. First, our area of study only covers two regions that have completely different rates of mothers who provide direct exclusive breastfeeding. As such, our samples do not represent the whole province. Although more samples may improve the findings, our results could provide a picture of what occurs in two different regions in terms of the cost of providing infant formula and/or CMF and factors that may influence the choice to DBF. Second, we used the CESD-R-10 to measure the mental health variable. This instrument can show depression symptoms but requires further examination to establish a firm diagnosis of depression. In addition, the instrument may have captured depression symptoms at certain periods of time (1 week) but cannot be generalized for a longer period, such as a year. However, we were able to portray the mothers who may experience depression symptoms during the period of exclusive breastfeeding. Recognizing the symptoms early is crucial for further consultation or treatment. Lastly, we did not include training/consultation related to the Baby-Friendly Hospital Initiative (BFHI) or Ten Steps to successful breastfeeding (Ten Steps) in our cost analysis as we have no information regarding the facility where the respondents gave birth. As the number of BFHI hospitals in Indonesia is still limited, it is less likely that the hospitals surrounding the area of our study are BFHI accredited. We found one study in Indonesia that estimate the cost of Ten Steps in a hospital in East Java province [52]. If we include all costs related to training or consultation on breastfeeding or risks of not doing so within the scope of the Ten Steps, this amounts to around US$800,000 (out of US$962,078) annually, using PPP factor in 2019 [52]. We do not, however, have the information of the average cost per person, so we cannot estimate the cost per mother for the purpose of our study. Additionally, the cost of BFHI or Ten Steps is rarely studied, especially in the Indonesian context. As such, more evidence is needed in more settings to have a better picture of how much it really costs per mother.

## Conclusions

The total cost of providing only commercial milk formula is 6-times higher than the cost of direct exclusive breastfeeding. The total cost and presence of severe depression symptoms are related to mother’s decision to not provide direct exclusive breastfeeding. This study shows that DBF is economically preferable to other methods and supports policies to increase the duration and quality of paid maternity leave as well as other maternity protection policies (e.g., lactation room, maternal cash transfer), and address the potential importance of a mother’s mental health to ensure successful direct exclusive breastfeeding.

## Data Availability

The datasets generated and analyzed in the current study are not publicly available due to patient confidentiality concerns but are available from the corresponding author upon reasonable request.

## Abbreviations

ASEAN: Association of Southeast Asian Nations.
CESD-R-10: Center for Epidemiologic Studies Depression Scale - Revised.
DBF: Direct Exclusive Breastfeeding.
IBF: Indirect Exclusive Breastfeeding.
CMF: Commercial Milk Formula.
GNI: Gross National Income.
IDR: Indonesia Rupiah.
PEB: Partial Exclusive Breastfeeding.
USD: United States Dollar.
